# Impact of augmented reality lessons on students’ STEM interest

**DOI:** 10.1186/s41039-016-0039-z

**Published:** 2016-07-07

**Authors:** Ying-Shao Hsu, Yuan-Hsiang Lin, Beender Yang

**Affiliations:** 1 Graduate Institute of Science Education, National Taiwan Normal University, No.88, Sect.4 Ting-Chou Rd., Taipei, 116 Taiwan; 2 Department of Electronic and Computer Engineering, National Taiwan University of Science and Technology, No.43, Sec. 4, Keelung Rd., Da’an Dist., Taipei, 106 Taiwan; 3 Saturn Imaging Inc., No.79, Sec. 1, Xintai 5th Rd., 14F-11, New Taipei City, 221 Taiwan

**Keywords:** STEM, Augmented reality, Authentic inquiry, STEM teaching, STEM education

## Abstract

In this paper, we explore the possibility of embedding augmented reality (AR) in authentic inquiry activities to contextualize students’ exploration of medical surgery, and investigate students’ perceptions of the AR lessons and simulators, and their Science, Technology, Engineering, and Mathematics (STEM) interest. Thirty-two senior high school students participated in the two AR lessons related to medical surgery, “laparoscopic surgery” and “cardiac catheterization.” The results showed that the students had positive perceptions of the AR lessons and simulators (overall mean = 4.1) after completing the two lessons. However, the authenticity of the simulators was perceived as the lowest ranking. In contrast, both the motivation and engagement triggered by the AR lessons were high, with most of the mean scores reaching 4.3. The AR lessons did evoke some students’ STEM interest as the survey results indicated that 12 students considered an STEM major in university. This study provides a possible solution for the alignment of instructional approaches (authentic inquiry), technology design (AR), and learning experience in developing STEM lessons.

## Background

Highly qualified Science, Technology, Engineering, and Mathematics (STEM) professionals will be needed for worldwide innovation and global economics (White House Office of Science and Technology Policy, 2012; Eisenhart et al., 2015). Regarding global economic competition, STEM education will help students develop their understanding of social issues (Garibay, 2015). In many countries, STEM is viewed as a means of developing the national economy and citizens’ scientific literacy (Nugent et al., 2015), but there seems to be decreasing readiness and motivation on the part of students to pursue STEM majors and careers (Osborne & Dillon, 2008). Educators need to ensure that human capital for STEM professions is well-trained for the workforce (Heitor, 2009). More STEM-focused schools which emphasize learning science, technology, engineering, and math have been established to improve US competitiveness, but some STEM schools are unlikely to be successful without systemic views on providing equal opportunities to learn STEM for culturally diverse students (Eisenhart et al., 2015). STEM-focused schools which have well-established partnerships with universities or foundations are more likely to gain more grants and extra resources to conduct surveys on understanding diverse students’ needs in order to improve the opportunity structure in schools.

The definition of STEM has not reached a solid conclusion, but it can be defined as a broad area encompassing many disciplines and epistemological practices (Lamb et al., 2015) or as using transdisciplinary knowledge and skills in solving real-world problems (Breiner et al., 2012; Labov et al., 2010; Sanders, 2009). Furthermore, “the extending definition of learning STEM is the acquisition of knowledge and skills through experience and study integrated through multiple lenses allowing for the appreciation of the encompassing complexity and crosscutting ideas across the STEM disciplines as a whole” (Lamb et al., 2015, p.410-411). It is noted that successful STEM learning is the total effect of the interaction of affect, cognition, and application of ideas (Oppezzo & Schwartz, 2013). Therefore, this study aims to develop integrated STEM lessons across different disciplines such as medical science, electronic engineering, and mathematics. The lessons allow students to apply crosscutting ideas in an authentic situation (e.g., role playing as an intern doctor). Students’ STEM interest is investigated in this study because interest creates the desire to learn content and skills within the STEM context.

An integrated STEM lesson needs to engage students in real-world situations and nurture their interest (Zeid et al., 2014). Even though real-world STEM situations are naturally integrated, classroom teachers seldom teach the integrated STEM contents. Therefore, it is suggested that the teaching practices for such lessons adopt manipulation and hands-on learning, cooperative learning, inquiry, problem-solving, and technology-enhanced learning (Stohlmann et al., 2012). In this study, a group of experts from different areas (e.g., science, engineering, technology, and education) collaborated to design integrated STEM lessons for developing the necessary competencies and practical skills of STEM professions. Showing the possibility of aligning different areas to solve problems can encourage students to work in groups of members with vastly different knowledge backgrounds. Therefore, this study intended to show the possibility of designing transdisciplinary learning examples in real-life situations for STEM teaching. The power of collaboration from different disciplines advances students’ conceptual integration across traditional disciplinary boundaries and the development of their expertise.

One of the major challenges of STEM education is to provide learning experiences that are meaningful to students with different needs (Talanquer, 2014). The visual intensity of STEM-integrated learning arises from representations and models of phenomena (Lamb et al., 2015), and there is solid evidence showing the correlation between STEM achievement and spatial visualization (Lubinski, 2010; Pruden et al., 2011; Wu & Shah, 2004). Spatial visualization reduces students’ cognitive load when engaging in STEM-related tasks through the additional cognitive channels to process data (Gonzalez-Castillo et al., 2012; Konstantinou et al., 2012). Therefore, we selected technology (such as augmented reality, AR) as a vehicle of manipulation and hands-on learning of integrating knowledge and practical skills in the form of spatial visualization.

AR offers a new form of interactivity between the physical and virtual worlds and enhances users’ perceptions of the real world (Kesim & Ozarslan, 2012). According to Wu et al.’s review (2013), AR allows students to develop important practices and has become one of the key emerging technologies in education. Although AR may present opportunities for teaching and learning, do students perceive an adequate level of realism when they are immersed in such a learning environment? How can researchers and educators work together to advance learning by aligning instructional approaches, technology design, and learning experience? This study proposes the possibility of embedding AR in authentic inquiry activities to contextualize students’ exploration of medical surgery. Especially, when bringing an engineering design into STEM teaching, AR technology can facilitate students’ manipulation of experiments in authentic contexts. The findings contribute to an understanding of how high school students perceive STEM lessons and the possible influence of STEM lessons on students’ career trajectories.

When do the critical experiences occur which influence students’ STEM interest and career choices? Some researchers claim that the STEM learning experiences at the elementary level facilitate students’ progress in STEM learning in their subsequent education (Dabney et al., 2013; Lamb et al., 2015; Maltese & Tai, 2010). With earlier STEM learning experiences, students likely become interested in STEM contents and in pursuing STEM-related careers. Furthermore, some research has pointed out that high school is the most critical time for career selection of scientists and engineers (Gayles & Ampaw, 2011; Maltese & Tai, 2011; Olson, 2009; Seymour & Hewitt, 1997; Spapiro & Sax, 2011; Sadler et al., 2012; Tyson et al., 2007). Experiences ranging from school coursework to summer camps may have a profound impact on high school students’ career choice (Riegle-Crumb et al., 2011). However, there is still a lack of literature in the effects of STEM-integrated programs on students’ affect, cognition, and skills. Therefore, STEM education is suitable to enact in high schools for promoting students’ STEM career interest.

## Methods

### Participants

This study aims to investigate high school students’ perceptions of AR and their STEM interest after they experienced two AR lessons which incorporated authentic inquiry activities for an exploration of medical surgery. A total of 32 grade 10 students (26 males and 6 females) from a private senior high school in Taipei city participated in this study, most of whom came from upper- and middle-class families. In these two AR lessons, the students were grouped into eight groups, each of which was provided with an Android tablet computer to interact with the surgery simulators through scanning QR codes.

### Procedures

**Fig. 1:**
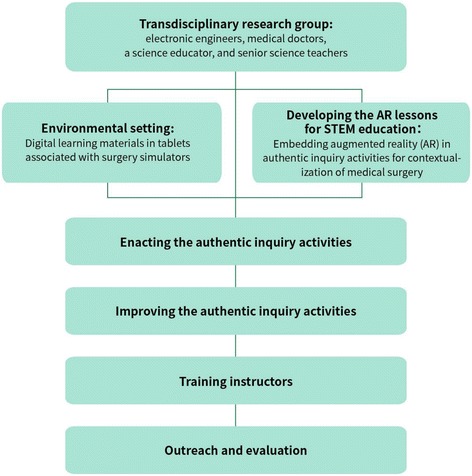
Research procedures

The students spent about 2.5 h participating in the intervention. First, they watched a 15-min video about how saving a valley from a disease attracted the cooperation of transdisciplinary experts. Then, the students worked in groups using tablets loaded with simulators to complete two AR lessons, laparoscopic surgery, and cardiac catheterization. One teaching assistant standing by each simulator guided each group of students to engage in the learning activities. After experiencing the AR lessons, the students completed a survey which was designed to elicit their perceptions of the AR lessons and simulators, their STEM interest, and their feedback on the lessons. This student feedback was to be used to elaborate the AR lessons and to develop training materials for instructors according to the suggested cycle of design-enact-evaluate-improve when implementing STEM education. A flow diagram summarizing the major procedures is shown in Fig. 1.

### The AR lessons

Current medical science is well-developed. One of the reasons is because there are sophisticated instruments for assisting doctors in the diagnosis and treatment of symptoms and diseases. Most of these instruments require the cooperation of doctors and engineers. Biomedical engineering is becoming popular because of this trend and the need for transdisciplinary cooperation. Since it is an interdisciplinary profession which must use engineering techniques to solve problems in biology and medicine, including diagnosis, monitoring, and providing therapy, this field has a real need for professionals with the ability to align the knowledge and techniques of the medical and engineering fields. Therefore, the personnel training for the biomedical engineering profession must include training in practical medical skills, the acquisition of medical knowledge, electronics, information systems, machinery, and practical engineering skills such as programming and electronic operations.

**Fig. 2:**
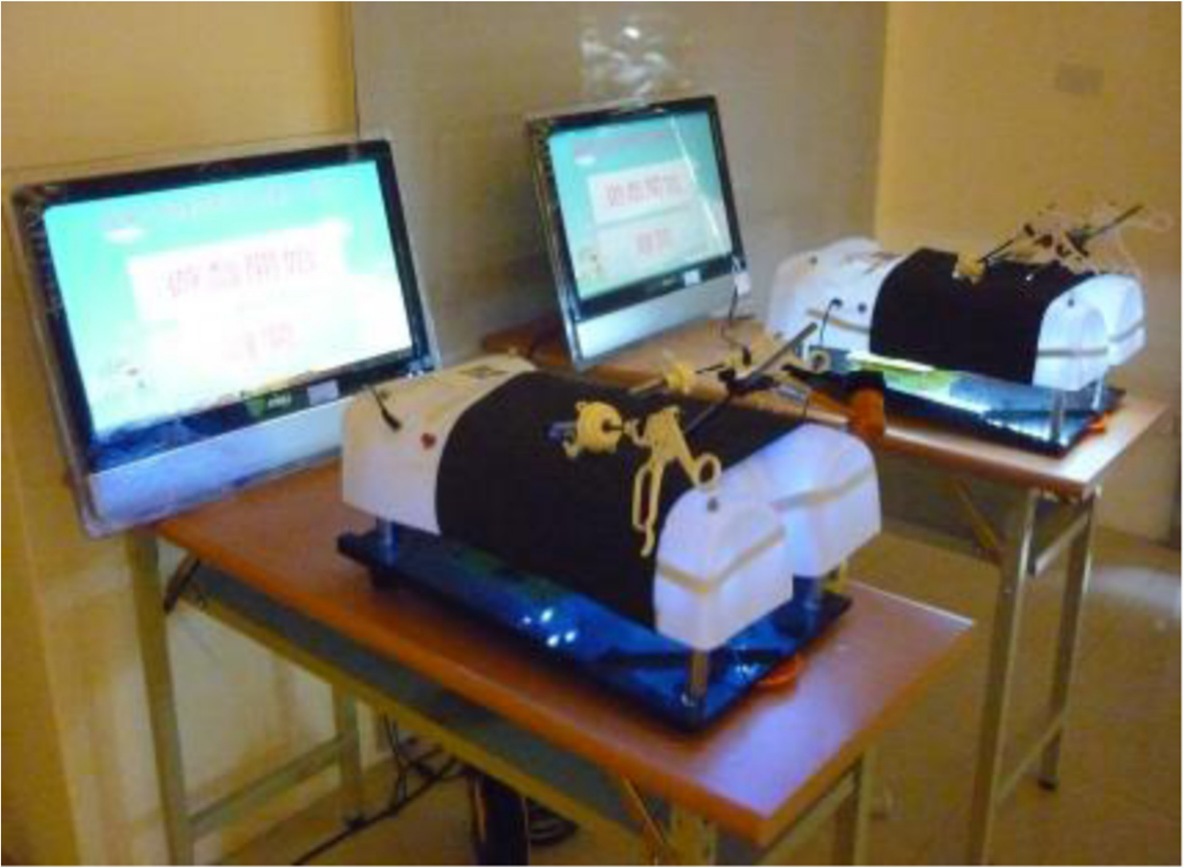
Laparoscopic surgery simulator

**Fig. 3:**
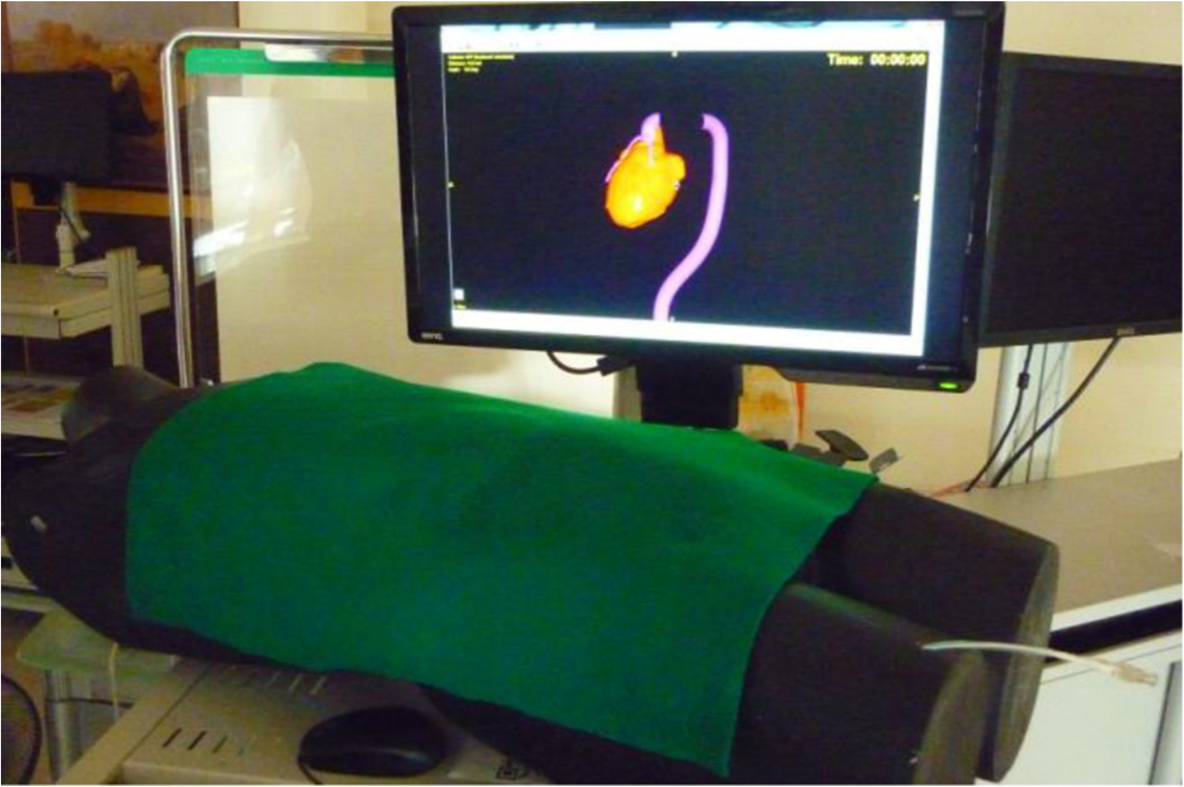
Cardiac catheterization simulator

Authentic inquiry refers to performing complex processes which scientists actually carry out (Chinn & Malhotra, 2001). Instead of the simple inquiry tasks seen in most science textbooks, authentic inquiry tasks allow students to interact with computer-simulated experiments or equipment so as to develop their inquiry skills. AR is a promising way to combine authentic contexts and simulated experiments for student exploration. Therefore, we incorporated AR with authentic inquiry to engage students in two surgical procedures, laparoscopic surgery, and cardiac catheterization. These activities were designed to facilitate the students’ experience of the diagnosis of symptoms and to operate the laparoscopic surgery and cardiac catheterization simulators (Figs. 2 and 3). These two lessons were designed to integrate innovative technologies in medical electronics and aimed to nurture the students’ STEM interest.

These two kinds of surgery are classified as minimally invasive surgery (MIS). MIS involves the latest technology and, compared with traditional open surgery, is widely used because it has the advantages of smaller incisions, less bleeding, and quicker recovery time. It is therefore frequently promoted in the medical field for its significant treatment benefits. MIS surgeons are considered as highly skilled medical professionals. In addition to the extensive knowledge required, they also need effective training tools to improve their skills. Therefore, training in operating skills, stabilization, and hand-eye coordination for surgeons is extremely important.

The simulators were developed by scientists, electronic engineers, and medical doctors from National Taiwan University, National Taiwan University of Science and Technology, and National Taiwan University Hospital. The engineers had to first understand the process of the surgery and the doctors’ needs in order to design the simulators. Sensors, circuit design, and programming techniques were used.

Cardiac catheterization is performed by inserting a catheter into the human vascular system (Baim & Grossman, 2006). Many vascular-related diseases can be treated with such minimal invasion. When the catheter reaches the target area, the treatment devices, such as angioplasty balloons or stents, can be initiated. The surgery is safe and effective. The advances in cardiac catheterization have made the traditional cardiac open surgery almost obsolete. In the clinical application, cardiac catheterization is regularly performed under angiography with radiation exposure. With the cardiac catheterization simulator, however, the inexperienced medical professional can be trained in a radiation-free environment. The simulator consists of a computer system and a human phantom (Fig. 3). A catheter can be inserted into the phantom’s upper leg. The simulator is also equipped with the catheterization operation technique to help the user understand the principle of catheter guidance without the pressure of performing a real operation. The technique includes, for example, when the contrast media should be released to increase vascular visibility and how to turn the catheter into the artery bypass. When the medical simulator is transformed into an AR simulator for STEM learning, these techniques are modified to become a problem-solving step. The vocal guidance and text message appear to trigger the student to solve the problem in order to advance to the next step of catheter guidance. The simulator can be operated in the edit mode to allow the instructor to design the catheter track for various clinical treatments, for example, coronary angioplasty and carotid artery stenosis treatment. The vocal and text guidance can be designed at the specific location in the track to simulate the training guidance from the surgeon master. The treatment options of the balloon and stents can also be selected in the catheterization simulator when the catheter reaches the target treatment location. In order to mimic the vascular system of the human anatomy, including the heart, arteries, and veins, a 3D mesh vascular system is installed within the simulator. The user can rotate this mesh freely in any orientation to simulate the fluorescent angiography image the surgeon sees during the surgery. The vascular track can be cut to reveal the location of the catheter tip in simulation. By operating the catheter inserted into the human phantom, the user can advance, retract, and rotate the catheter. The real-time position of the catheter tip can be visualized on the screen in synchronization. A timer is installed to record the duration of the operation, providing the performance of the user to the instructor for evaluation purposes.

In the context of medical training, users may be attracted by the real-world scenario presented by these AR surgery simulators. While originally designed for the training of medical majors, in this study, the simulators were adopted for the purpose of letting the students learn some medical knowledge, while also allowing them to understand how to set up a simulator. While experiencing the surgery simulation, students can start to learn the pros and cons of minimally invasive surgery and traditional surgery and can repeatedly simulate the actual operation so as to experience the situation of doctors performing surgery. Such practical examples can arouse students’ interest, and they may thus come up with better ways to solve some of the problems encountered in the field of medicine.

**Fig. 4:**
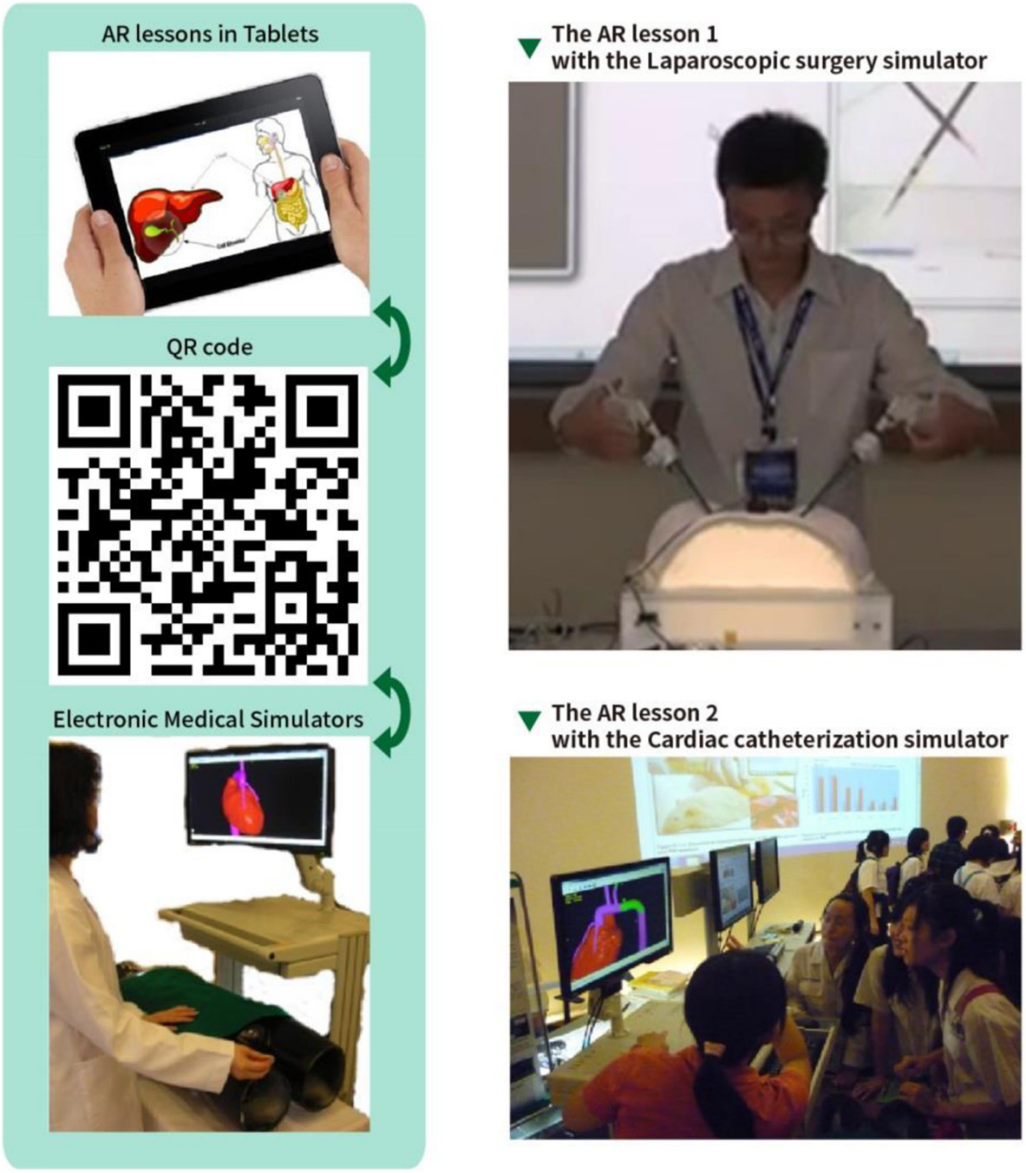
Designs of authentic inquiry activities with tablets and surgery simulators

For the purpose of outreach, senior high school teachers in Taipei city and a science educator from National Taiwan Normal University were invited to design authentic inquiry activities introducing medical practical skills to senior high school students to help them explore the possibility of a future career in STEM. A group of electronic engineers helped develop the AR functions with simulators and e-learning materials on tablets. A total of 32 students were grouped into eight groups, each of which was provided with an Android tablet computer to interact with the simulators using the scanning function on the tablet (Fig. 4). The tablet computer delivered an authentic context of a patient’s symptom, and the students were required to diagnose the possible disease using the data such as X-ray images and electrocardiograms on the tablet. Then, the students used the simulator to help the patient recover from the disease. We applied the role-play technique in these activities whereby the students were told that they were surgery interns who were required to learn practical surgical skills.

For the laparoscopic surgery lesson, the students were told that they were to role-play surgery interns. Each group of students worked with a tablet and the simulator. First, a video clip on the tablet showed a patient describing his symptoms, and the students were required to diagnose the possible disease after checking the X-ray images. Second, some surgery options were shown to the students from which they were required to select the most appropriate procedure for treating the disease. Third, the students used the scanning function to activate the simulator and operated the laparoscopic surgery simulator which displays 2D images for promoting practical skills (Fig. 2). Finally, the students needed to reflect on the pros and cons of laparoscopic surgery and to offer their opinions regarding improvements to such surgery.

The cardiac catheterization lesson was developed following a similar procedure to that of the laparoscopic surgery lesson. The students role-played surgery interns to diagnose a patient’s disease from the electrocardiogram on the tablet. Then, they used the scanning function to activate the simulator and carried out angioplasty by operating a catheter in the simulator. A 3D heart image was displayed synchronously on the computer to indicate the location of the catheter when the students moved it in the simulator. For some critical points, the computer provided a doctor’s advice to help the students overcome their difficulties operating the catheter. At the end, the students needed to answer some questions related to cardiac catheterization.

### Questionnaire

After the AR lessons, the students were asked to complete a questionnaire according to their user experience. This questionnaire included three parts: STEM interest, perceptions of the AR lessons and simulators, and feedback about the lessons. The first part of the questionnaire was the student’s STEM interest indicated by the potential choice of major in university. The last part of the questionnaire consisted of perception items which were originally developed to investigate students’ experience of working in an authentic electronic learning environment. We revised the items of the user experience questionnaire from the study of Chang et al. (2010) who used the constructs referring to the study of Gulikers et al. (2006). Two designs in our learning system were examined including the AR lessons and simulators. Each of them consisted of nine Likert-type items with a five-point scale to examine three constructs: authenticity, engagement, and learning motivation. As Table 1 shows, the reliability of the questionnaire ranges from 0.63 to 0.97 for the constructs, but the overall reliabilities for the AR lessons and simulators reach 0.82, 0.89, 0.81, and 0.92, respectively. The last part of the questionnaire is an open-ended question about their overall opinions of the AR lessons.

**Table 1:** Reliability of questionnaire (Cronbach’s alpha)

		Laparoscopic surgery	Cardiac catheterization
AR lessons	Authenticity	0.80	0.69
	Engagement	0.78	0.63
	Motivation	0.87	0.87
	Total	0.82	0.81
Simulators	Authenticity	0.84	0.87
	Engagement	0.97	0.94
	Motivation	0.71	0.68
	Total	0.89	0.92

## Results and discussion

Tables 2 and 3 show the descriptive statistic results of students’ perceptions of the two AR lessons and simulators in laparoscopic surgery and cardiac catheterization, respectively. Overall, the means of authenticity in the AR lessons and simulators are lower than the means of engagement and learning motivation. Furthermore, we examined the significances of the differences among these constructs (authenticity, engagement, and learning motivation) using Kendall’s W test since not all of the measurements reached the assumption of normal distribution. The results of Kendall’s W test on student perceptions of the AR lessons and simulators are shown in Table 4. No significant difference was found in the AR instructional design of laparoscopic surgery (*χ* ^2^ = 3.77, *p* < 0.15), but there was a significant difference found in the laparoscopic surgery simulator (*χ* ^2^ = 30.66, *p* < 0.001). The students’ perceived engagement and learning motivation regarding the laparoscopic surgery simulator was higher than its perceived authenticity. In contrast, there were significant differences in both AR instructional design (*χ* ^2^ = 8.53, *p* < 0.01) and surgery simulator (*χ* ^2^ = 31.19, *p* < 0.001) of the cardiac catheterization lesson. The students’ perceived engagement in the AR instructional design for cardiac catheterization was higher than their perceived authenticity, and perceived engagement and learning motivation for the cardiac catheterization simulator were higher than perceived authenticity.

**Table 2:** Summary of students’ perceptions of the laparoscopic surgery lesson and the simulator

Authentic inquiry activities on tablets: laparoscopic surgery		
Item	Mean	S.D.
Authenticity	4.0	0.7
I feel what I am seeing is connected to real-life situations when I use the tablet to learn laparoscopic surgery.	4.1	0.7
I feel that the digital learning materials of the laparoscopic surgery lesson are authentic.	3.9	0.7
The learning activities in the laparoscopic surgery lesson are related to real-life situations.	4.1	0.8
Engagement	4.3	0.7
I concentrate on learning activities in the laparoscopic surgery lesson.	4.6	0.5
I was involved in the learning activity because of the virtual character and the virtual scene.	4.1	0.7
The virtual character and the scene in the laparoscopic surgery lesson facilitated my completion of the learning activity.	4.1	0.8
Learning motivation	4.1	1.1
I like to play the role (an intern doctor) in the virtual laparoscopic surgery scene.	4.0	1.1
I like to learn with the digital material of the laparoscopic surgery lesson.	4.2	1.1
I want to learn the laparoscopic surgery lesson again.	4.2	1.2
Total	4.1	0.8
The laparoscopic surgery simulator		
Item	Mean	S.D.
Authenticity	3.6	1.1
I feel the laparoscopic surgery simulator is very real.	3.8	1.0
I feel that the laparoscopic surgery simulator and the learning content shown on the monitor are coordinated.	3.4	1.2
The learning activities with the laparoscopic surgery simulator are related to real-life situations.	3.7	1.0
Engagement	4.3	1.1
I concentrate on the learning activities with the aid of operating the laparoscopic surgery simulator.	4.3	1.1
I enjoy the learning activities in the tablet because of interacting with the laparoscopic surgery simulator.	4.2	1.1
I concentrate on operating the laparoscopic surgery simulator.	4.3	1.1
Learning motivation	4.3	0.7
I like to operate the laparoscopic surgery simulator.	4.3	0.8
I become more interested in medical information because of the laparoscopic surgery simulator.	4.2	0.7
I want to use the laparoscopic surgery simulator to learn medical topics again.	4.5	0.6
Total	4.1	1.0

**Table 3:** Summary of students’ perceptions of the cardiac catheterization lesson and the simulator

Authentic inquiry activities on tablets: cardiac catheterization		
Item	Mean	S.D.
Authenticity	3.9	0.7
I feel what I am seeing is connected to real-life situations when I use the tablet to learn cardiac catheterization.	3.9	0.7
I feel that the digital learning materials of the cardiac catheterization lesson are authentic.	3.9	0.6
The learning activity in the cardiac catheterization lesson is related to real-life situations.	3.8	0.8
Engagement	4.2	0.7
I concentrate on learning activities in the cardiac catheterization lesson.	4.3	0.6
I was involved in the learning activity because of the virtual character and the virtual scene.	4.2	0.6
The virtual character and the scene in the cardiac catheterization lesson facilitated my completion of the learning activity.	4.1	0.8
Learning motivation	4.0	1.1
I like to play the role (an intern doctor) in the virtual cardiac catheterization scene.	3.8	1.0
I like to learn with the digital material of the cardiac catheterization lesson.	4.2	1.0
I want to learn the cardiac catheterization lesson again.	4.1	1.2
Total	4.0	0.8
The cardiac catheterization simulator		
Item	Mean	S.D.
Authenticity	3.7	1.0
I feel the cardiac catheterization simulator is very real.	3.5	1.0
I feel that the cardiac catheterization simulator and the learning content shown on the monitor are coordinated.	3.9	1.1
The learning activities with the cardiac catheterization simulator are related to real-life situations.	3.6	1.0
Engagement	4.3	1.0
I concentrate on the learning activities with the aid of operating the cardiac catheterization simulator.	4.3	1.0
I enjoy the learning activities on the tablet because of interacting with the cardiac catheterization simulator.	4.3	1.0
I concentrate on operating the cardiac catheterization simulator.	4.4	1.0
Learning motivation	4.2	0.6
I like to operate the cardiac catheterization simulator.	4.2	0.6
I became more interested in medical information because of the cardiac catheterization simulator.	4.1	0.6
I want to use the cardiac catheterization simulator to learn medical topics again.	4.4	0.7
Total	4.1	0.9

**Table 4:** Summary of Kendall’s W test on students’ perceptions of the AR lessons and the simulators

Lesson	Equipment	Constructs	Mean	SD	Mean rank	*W*	*χ*2	*df*	*p*	Post hoc
Laparoscopic surgery	Tablets	Authenticity	12.09	1.91	1.75	0.06	3.77	2	<0.15	
		Engagement	12.81	1.66	2.13					
		Motivation	12.38	2.93	2.13					
	Simulators	Authenticity	10.88	2.79	1.31	0.48	30.66	2	<0.001	Moti > Authen (*p* < 0.001)
		Engagement	12.81	3.11	2.48					Enga > Authen (*p* < 0.001)
		Motivation	12.88	1.68	2.20					
Cardiac catheterization	Tablets	Authenticity	11.59	1.66	1.66	0.13	8.53	2	<0.01	Enga > Authen (*p* < 0.018)
		Engagement	12.59	1.54	2.25					
		Motivation	12.03	2.87	2.09					
	Simulators	Authenticity	11.03	2.74	1.30	0.49	31.19	2	<0.001	Moti > Authen (*p* < 0.001)
		Engagement	12.97	2.80	2.47					Enga>Authen (*p* < 0.001)
		Motivation	12.69	1.45	2.23					

*Note*: In the post hoc column, Moti means motivation, Authen means authenticity, and Enga means engagement

Students’ STEM interest was indicated by their potential choice of major in university. The results showed that there were 12 students considering STEM majors in university, 11 considering medical-related departments, 3 considering electronics, 4 considering science-related departments, and 2 considering social science as their major. Since we only use the post-lesson survey to investigate students’ perception of an AR inquiry lesson, it is difficult to determine the extent to which students’ choice of STEM-relative majors in university was influenced. It limits the implication of this result.

In addition, students’ feedback on the AR lessons from an open-ended question included gaining a sense of being a doctor and surgery procedure (12 students), comments on authenticity and design (13 students), promoting medical knowledge (4 students), understanding the importance of aligning medicine and engineering for advancing health issues (4 students), and evoking STEM career interest (4 students). The students thought that the learning experiences from these two AR lessons inspired them about the progress of medical technology and the importance of transdisciplinary work for the innovation and creativity needed in future workplaces. They reported that the AR lessons motivated them to choose STEM-related majors in university because the video showed an example of aligning electronics and medical science to save people, the AR lessons displayed the innovation of medical science with the aid of electronics and mechanics, and the learning experience led them to think of other possibilities of transdisciplinary invention.

Even though the students’ responses to the open-ended questions showed that some thought the simulators displaying the surgery process were clear and related to real-life situations, increased their medical knowledge, and represented the characteristics of being a doctor through experiencing how to operate them, they also provided suggestions about the simulators including reducing the response time between the operating equipment and the computer and adding more diagnosis processes of patients’ symptoms into the learning activities. Although we used very similar equipment to that used in actual medical surgery, the students still perceived that the simulators were not as realistic as they might have been. More improvements can be made to increase the realism of simulators such as connecting surgical equipment to a human model which can react to students’ operation synchronously through sensors. In the future, virtual reality (VR) technology can also be used to simulate more realistic surgical procedures to let users feel that the simulation is truer to life and allowing them to have more interactions with the surgery simulators (Sinitsky et al., 2012; Combs, 2010). Another direction is to build a mixed physical/virtual simulator. Not only would it have the dynamic effect of virtual reality but it would also have a tactile feedback component, becoming more realistic and offering the user richer experiences (Carbone et al., 2011).

The AR lessons provided opportunities for high school students to learn integrated STEM contents and practical skills of medical surgery. Such learning experiences could be critical in nurturing students’ STEM interest and motivating them to select STEM-related majors in university. However, how students’ competencies in STEM developed were not measured in this study. There was no solid evidence to show how well students’ competencies in STEM developed during the two AR lessons. It is therefore suggested that an instrument be designed to detect students’ STEM competencies for future research. Also, are there other instructional approaches for advancing students’ STEM competencies? Besides well-designed AR lessons with authentic inquiry activities, teachers are also encouraged to apply an engineering design process method in STEM teaching. This method, which combines systematic structure, organized tools, proper resources, and hands-on and real-world projects, includes several steps: starting with a problem, conducting research, brainstorming possible solutions, choosing the best solution that satisfies the constraints, designing and building a prototype, testing the prototype, redesigning, and communicating (Zeid et al., 2014). In such an instructional approach, students would perform STEM competencies autonomously so that teachers will have a chance to observe their progress in STEM competencies, attitudes, and skills. The engineering design process method is effective for STEM teaching in high school, can bridge the disconnection between theory and application in STEM classes, and would provide an opportunity for students to gain insight into STEM careers (Zeid et al., 2014).

Another challenge for STEM education is to train teachers in how to teach integrated STEM in schools. For most schools, it is not common to teach transdisciplinary topics or to apply effective instructional approaches to connect theory and application in STEM such as authentic inquiry and the engineering design process method. There is a lack of research focusing on teachers’ training for STEM education. More studies are needed to identify the challenges and effective strategies for STEM teaching and how to help teachers enact integrated STEM lessons in the classroom.

## Conclusions

We embedded AR in authentic inquiry activities so that students could experience when and how to carry out certain medical surgical procedures. The overall average score of student perceptions of the AR lessons and simulators were higher than 4.0, except for the authenticity of the cardiac catheterization simulator. We found evidence that embedding AR in authentic inquiry promotes students’ engagement and motivation in developing the practical skills for medical surgery and inspired them to select STEM-related majors in university. Since high school experiences are the most critical for students’ STEM career choice (Olson, 2009; Riegle-Crumb et al., 2011), AR lessons provide authentic inquiry activities with surgery simulators to display the alignment between medicine and electronics as an example of transdisciplinary innovation. This learning experience nurtured the students’ STEM interest and possibly had a profound impact on their university major selection. It is strongly suggested that more STEM lessons be developed to motivate and engage students in transdisciplinary learning with new technology and authentic situations. More research is needed to understand how to apply different instructional approaches in STEM teaching and to examine how students’ development of valued competencies, STEM career interest, and life-long learning abilities can be promoted by STEM teaching.
